# Retraction Note: The oncogenic Golgi phosphoprotein 3 like overexpression is associated with cisplatin resistance in ovarian carcinoma and activating the NF-κB signaling pathway

**DOI:** 10.1186/s13046-025-03344-4

**Published:** 2025-02-26

**Authors:** Shanyang He, Gang Niu, Jianhong Shang, Yalan Deng, Zhiyong Wan, Cai Zhang, Zeshan You, Hongwei Shen

**Affiliations:** 1 Department of Obstetrics and Gynecology, The First Affiliated Hospital, Sun Yat-Sen University, Zhongshan Second Road 58, Guangzhou, 510700 People’s Republic of China; 2 Department of Ultrasonic Medicine, Fetal Medical Center, the First Affiliated Hospital, Sun Yat-Sen University, Guangzhou, People’s Republic of China

**Retraction Note** **:** **J Exp Clin Cancer Res 36, 137 (2017)**

**https://doi.org/10.1186/s13046-017-0607-0**

The Editor-in-Chief has retracted this article. Several image integrity concerns were brought to the attention of the publisher after publication, including:

- In figure 2B, FASC scatter plots appear to contain duplications between the GOLPH3L sh#1 SKOV3, GOLPH3L sh#2 SKOV3 and GOLPH3L sh#2 A2780 plots
- In figure 3F, the top of image A2780 vector-Active Caspase 3 appears to contain overlap with the bottom of A2780 GOLPH3L- Active Caspase 3
- In figure 4E, there appear to be high similarities between panel α-Tubulin - SKOV3 - control/GOLPH3L-2h#1- control/GOLPH3L-2h#2 and figure 5A α-Tubulin in another article published earlier by different authors [1].

The authors were not able to provide a satisfactory explanation for these concerns and described repeated issues with data labelling and storage practices. Therefore the Editor has lost confidence in the data and conclusions of this article.

Author Shengyang He disagrees with this retraction. All other authors have not responded to correspondence regarding this retraction.
